# Body mass index and waist/height ratio for prediction of severity of coronary artery disease

**DOI:** 10.1186/1756-0500-7-246

**Published:** 2014-04-17

**Authors:** Khandker MD Nurus Sabah, Abdul Wadud Chowdhury, HI Luftur Rahman Khan, ATM Hasibul Hasan, Serajul Haque, Shomsher Ali, Shamima Kawser, Nur Alam, Gaffar Amin, S M Ear E Mahabub

**Affiliations:** 1 Department of Cardiology, Dhaka Medical College Hospital, Dhaka, Bangladesh; 2 Department of Neurology, Dhaka Medical College Hospital, Dhaka, Bangladesh; 3 Department of Microbiology, Delta Medical College Hospital, Dhaka, Bangladesh

**Keywords:** BMI, Waist to height (WHt) ratio, Coronary artery disease (CAD)

## Abstract

**Background:**

To determine whether waist-to-height ratio correlates with coronary artery disease (CAD) severity better, than the body mass index (BMI) as assessed by coronary angiography in Bangladeshi population.

**Methods:**

This cross sectional study was done on patients in Department of Cardiology in DMCH and those referred in the cath-lab of the Department of Cardiology for CAG during November 2009 to October 2010 involving 120 patients. They were divided into group-A (with coronary score ≥7) and group-B (coronary score <7) depending on Gensisni score.

**Result:**

There were no statistically significant difference regarding the distribution of age, sex and clinical diagnosis and parameters between the two groups. The mean age of patients was 51.7 ± 8.2 years and 48.8 ± 9.1 years in Group A and Group B respectively with a male predominance in both the groups. Patients in group A had higher BMI ≥25 and waist to height ratio (≥0.55) than Group B which showed a statistically significant association (p < 0.001). Though a significant positive correlation (r = 0.296, p = 0.006) was observed between BMI and Coronary artery disease score in group A patients, scenario was reverse fro group B (r = 0.076, p = 0.659). The statement was also true for Waist-to-height ratio and Waist-to-height ratio with BMI. Multivariate analysis also yeilded that a patient with BMI ≥25 kg/m^2^ and waist-to height ratio of ≥0.55 are 3.06 times and 6.77 times, more likely to develop significant coronary artery disease respectively.

**Conclusion:**

The waist-to-height ratio showed better correlation with the severity of coronary artery disease than the BMI.

## Background

Coronary heart disease (CHD) caused about one of every five deaths in the United States in 2005. It is the largest single killer of American males and females [1]. National data on incidence and mortality of coronary heart disease are few in Bangladesh. The prevalence of coronary heart disease was estimated as 3.3/1000 in 1976 and 17.2/1000 in 1986 indicating 5 folds in the disease in 10 years [2]. In 1975, the incidence of ischemic heart disease (IHD) in Bangladesh was reported to be 3.3 per thousand which subsequently increased to 14 per thousand in 1985 [3]. As quoted by Malik, WHO reported the incidence of IHD in Bangladesh as 11 percent among all the cardiac disorders [4]. Among the hospitalized patients in National Institute of Cardiovascular Disease (NICVD), Dhaka, IHD were 56 percent of all cardiac problems [5].

Traditionally there are some conventional risk factors for CAD e.g. age, male sex, positive family history, hypertension, smoking, hyperlipidaemia, metabolic syndrome, diabetes, lack of exercise, obesity, and some emerging risk factors, e.g. C-Reactive Protein, Homocysteine, Fibrinogen etc. [6]. The body mass index, waist circumference, waist/hip ratio, waist/height ratio and skin fold thickness, all are clinical tools for evaluatation of obesity and fat distribution. Among these, body mass index is the best studied predictor of risk of complications related to obesity [7]. As a limitation, some people within normal BMI range may have excessive central fat accumulation and elevated metabolic risks [8] and there are evidences which link central (visceral or intra-abdominal) obesity more strongly than peripheral fat distribution with the subsequent development of cardiovascular disease and maturity-onset diabetes [9]. As central fat distribution is considered more atherogenic than peripheral obesity, much attention has been focused on methods that can evaluate central obesity [10]. However, the ratio of waist circumference to height ratio has been proposed as a better predictor of cardiovascular risk [11], mortality [12] and intra-abdominal fat distribution [13]. The waist-to-height ratio was first used in the Framingham Study [14]. Several studies of children [15] and adults [16] have concluded that this ratio is more strongly associated with cardio vascular risk factors than the body mass index (BMI; in kg/m^2^). In a population-based study from Hong Kong, this ratio has been most strongly associated with cardiovascular risk with a suggested cutoff value of 0.5 for Asian population [17]. However no study has yet been done in Bangladeshi IHD population. This study was conducted to investigate whether BMI or WHtR is a better predictor for CAD in Bangladeshi IHD population.

## Methods

This cross sectional study was carried out among 120 patients admitted with ishchemic heart disease (IHD) at Department of Cardiology, Dhaka Medical College & Hospital (DMCH) from November 2009 to October 2010. Patient selection was done through purposive sampling technique.

The body height was taken in the standing position without shoes. Weight was measured similarly without shoes and heavy dresses immediately before coronary angiography (CAG). Waist circumference (WC) was measured at the mid-point between the distal border of the ribs and the top of the iliac crest with subjects standing at the end of a normal expiration. Body mass index (BMI) was calculated by dividing body weight in kilograms by the square of body length in meters (kg/m^2^). Waist height ratio (WHtR) was calculated by dividing waist circumference by height (cm). All cases were grouped according to their waist height ratio value [10,18]. All consecutive patients who underwent coronary angiography for IHD fulfilling the selection criteria were included to participate in the present study.

During the first visit, a complete history regarding the reason for angiography request, past medical history and medications (including weight-reducing drugs) were obtained. Weight was recorded by a standard medical scale. All participants were weighed with light clothing and without shoes. Height was measured while standing with four parts (heels, buttocks, back and head) touching the stadiometer, heels together and head in Frankfurt plane. BMI was calculated as weight/height^2^ (kg/m^2^) and WHtR as WC/height. BMI ≥ 25 kg/m^2^ was consided as overweight and ≥ 30 kg/m^2^ was considered obese. The criterion of abdominal obesity was defined as WHtR ≥0.55 [10]. Subsequently, participants were divided into two groups based on WHtR which was normal <0.55 and abnormal ≥0.55 and two groups based on BMI that was normal (<25 kg/m^2^) and over weight (≥25 kg/m^2^) for better evaluation of their roles in the prediction of coronary artery disease (CAD).

CAD was scored after coronary angiography according to the following criteria: (1) the number of atherosclerotic main coronary arteries (0–3); (2) the number of atherosclerotic segments of main coronary arteries (0–9, each coronary artery has 3 segments), and (3) the severity of atherosclerotic stenosis of main coronary arteries (0 = no stenosis; 1 = stenosis <50.0%; 2 = stenosis 50.0–75.0%, and 3 = stenosis >75.0%). The total coronary artery score was assessed by the above criteria; between 0 and 21 [19].

### Study group

•Scores ≥7 was significant CAD considered as group A and

•scores <7 was without significant CAD considered group B [19].

### Inclusion criteria

1. Patients with IHD (Chronic stable angina, unstable angina, acute ST segment elevated myocardial infarction, none ST segment elevated myocardial infarction) undergoing first time coronary angiography for CAD detection in the Department of Cardiology DMCH.

### Exclusion criteria

1. Patients reffered for CAG other than CAD (congenital heart disease and valvular heart disease.

2. Patients with history of previous Coronary artery bypass grafting (CABG) or percutaneous coronary intervention (PCI).

3. Those who had participated in weight-reducing programs (including diets) or received related medications.

4. Any systemic infection or any other serious comorbid condition.

5. Unwilling to give concent.

### Ethical consideration

Prior to the commencement of this study, the research protocol was approved by the Research Review Committee of Department of Cardiology and the Ethical Committee of DMCH, Dhaka. The aims and objective of the study along with its procedure, alternative diagnostic methods, risk and benefits were explained to the patients in easily understandable local language and then informed consent was taken from each patient.

### Statistical analysis

Statistical package for social science (SPSS) 16.0 was used in statistical analysis of the data. χ^2^-test was used in the comparison of quantitative data. Student’s t-test, multivariate logistic regression was used in the comparison of quatitative and qualitative data. The significance of the results as determined in 95.0% confidence interval and a value of P < 0.05 was consider to be statistically significant.

## Results

This cross sectional study involved 120 parients who were divided into Group-A (coronary score ≥7) and group-B (coronary score <7). It was observed that most (41.7%) of the patients having significant CAD (group A) belonged to 6^th^ decade whereas a half (50.0%) of the patients belonged to 5^th^ decade among those without significant CAD (group B). The mean age was 51.7 ± 8.2 years and 48.8 ± 9.1 years in group A & B respectively. Male were predominant in both groups (81.0% and 83.3%) with a male female ratio of 4.5:1 (Table 1). There was no statistically significant difference regarding age distribution, sex ratio and clinical diagnosis between these two groups (Table 2).

**Table 1:** Demographic profile of the patients (n = 120)

**Parameter**	**Group A (n = 84)**		**GroupB (n = 36)**		**P Value**
	**n**	**%**	**n**	**%**	
**Age in years**					
31-40	9	10.7	6	16.7	
41-50	25	29.8	18	50	
51-60	35	41.7	8	22.2	
61-70	15	17.9	4	11.1	
<50	34	40.5	24	66.7	
>50	50	59.5	12	33.3	
Mean ± SD	51.7 ± 8.2		48.8 ± 9.1		0.089 ns
**Sex**					
Male	68	81	30	83.3	
					0.757 ns
Female	16	19	6	16.7	

Group A: Significant CAD (Coronary artery disease score ≥7).

Group B: without significant CAD (Coronary artery disease score <7).

NS = Non Significant.

P value reached from unpaired t-test.

**Table 2:** Comparison of the study groups according to clinical diagnosis (n = 120)

**Clinical diagnosis**	**Group A (n = 84)**		**Group B (n = 36)**		**P Value**
	**n**	**%**	**n**	**%**	
Stable angina	28	33.3	14	38.9	0.558^ns^
Unstable angina	11	13.1	4	11.1	0.763^ns^
Acute STEMI	15	17.9	5	16.7	0.592^ns^
Non STEMI	3	3.6	1	2.8	0.824^ns^
OMI	27	32.1	11	30.6	0.863^ns^

NS = Not significant.

P value reached from chi square test.

**Figure 1:**
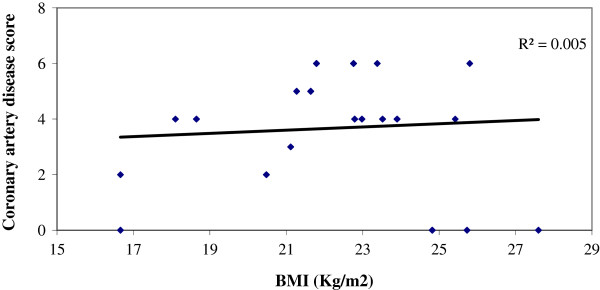
Relation between BMI with Coronary artery disease score, in group without significant CAD (Group B).

**Figure 2:**
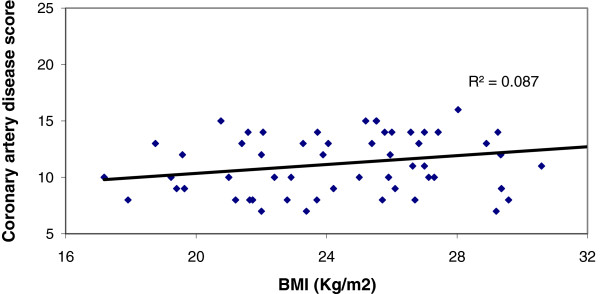
Relation between BMI with Coronary artery disease score, in patients with in group with significant Cad (Group A).

**Figure 3:**
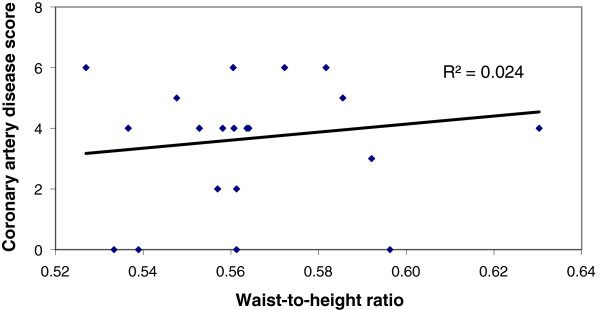
Relation between Waist-to-height ratio with Coronary artey disease score in group without significant CAD (Group B).

**Figure 4:**
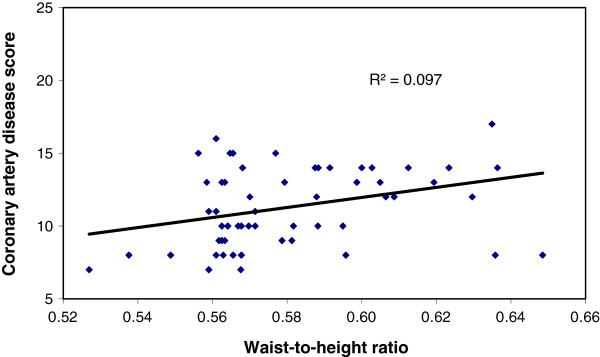
Relation between Waist-to-height ratio with Coroary artey disease score in group with significant CAD (group-A).

**Figure 5:**
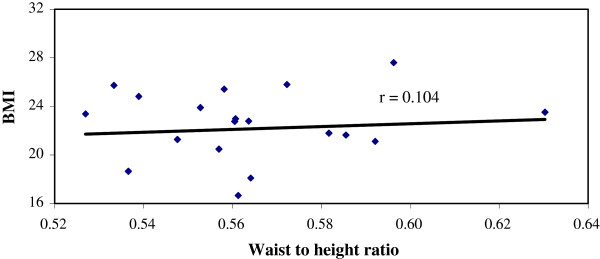
Relation between Waist-to-height ratio with BMI in group without significant CAD (group-B).

**Figure 6:**
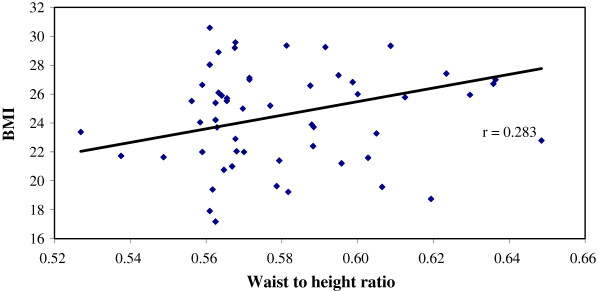
Relation between Waist-to-height ratio with BMI in group with significant CAD (group-A).

Significantly large number of patients in Group A had BMI ≥25 than those of Group B, (47.6% vs 11.1%), p < 0.001) (Table 3). Similarly, the percentage of patients with waist to height ratio (≥.55) was higher in group A (86.9% vs 47.2%) than group B (Table 4). We have found a positive correlation (r = 0.296, p = 0.006) between BMI and Coronary artery disease score among patients within the group with significant CAD (GroupA) But there was no correlation (r = 0.076, p = 0.659) between BMI and Coronary artery disease score among patients of Group B, (Figures 1 and 2). Similarly, significant positive correlation was found between Waist-to-height ratio (r = 0.311, p = 0.004), Waist-to-height ratio with BMI (r = 0.283, p = 0.001) in group with significant CAD (group-A), but not for group B (r = 0.155, p = 0.366), (r = 0.104 p > 0.05) (Figures 3, 4, 5, 6). Multivariate analysis also yeilded that a patient with BMI ≥25 kg/m^2^ and waist-to height ratio of ≥0.55 are 3.06 times and 6.77 times, more likely to develop significant coronary artery disease respectively (Table 5).

**Table 3:** Comparison of BMI between two groups (n = 120)

**BMI**	**Group A (n = 84)**		**Group B (n = 36)**		**OR**	**95% CI**	**chi/t value**	**P Value**
	**n**	**%**	**n**	**%**				
BMI ≥25	40	47.6	4	11.1	7.27	2.18-26.7	14.46	^a^0.001^s^
BMI <25	44	52.4	32	88.9				
Mean ± SD	24.6	±4.1	22.2	±2.6			3.240	^b^0.001^s^
Range (Min-Max)	(17.2	−46.2)	(16.7	−27.6)				

S = Significant.

^a^P value reached from chi square test.

S = Significant.

^b^P value reached from unpaired t-test.

**Table 4:** Comparison of waist to height ratio between study groups (n = 120)

**Waist-to height ratio**	**Group A (n = 84)**		**Group B (n = 36)**		**OR**	**95% CI**	**chi/t value**	**P Value**
	**n**	**%**	**n**	**%**				
≥0.55	73	86.9	17	47.2	7.42	2.73-20.55	21.16	^a^0.001^s^
<0.55	11	13.1	19	52.8				
Mean ± SD	0.584	±0.051	0.563	±0.021			2.381	^b^0.001^s^
Range (Min- Max)	(0.488	−0.970)	(0.533	−0.630)				

S = Significant.

^a^P value reached from chi square test.

S = Significant.

^b^P value reached from unpaired t-test.

**Table 5:** Multivariate predictors of significant CAD with risk factors (n = 120)

	**Crude OR**	**95% CI**		**P value**	**Adjusted**	**95% CI**		**P value**
		**Lower**	**Upper**		**OR**	**Lower**	**Upper**	
BMI	7.27	2.18	26.7	0.001^s^	3.06	1.057	8.870	0.039
Waist-to height ratio	7.42	2.73	20.55	0.001^s^	6.77	1.932	23.692	0.003
Smoking	9.29	2.9	31.01	0.001^s^	0.30	.103	.870	0.027
Hypertension	3.9	1.47	10.45	0.001^s^	0.23	.073	.736	0.013
Diabetes-mellitus	5.07	2.02	12.97	0.001^s^	0.72	.251	2.040	0.531
Family history	16.33	5.59	49.7	0.001^s^	0.44	.132	1.433	0.171
Dyslipidemia	16.82	5.81	50.53	0.001^s^	0.41	.140	1.196	0.102

S = Significant, NS = not significant.

## Discussion

In our study coronary artery disease had significant positive correlation with both BMI and waist-to-height ratio. Among these two indices, positive correlation was stronger with waist-to-height ratio and the severity of coronary artery disease. Studies, including this one have shown their good predictive value for assessing the severity of CAD. The waist/height ratio is proving to have a strong association with cardiovascular risk factors in Asian population [20]. So we wanted to evaluate it on our population. The mean age was not statistically different between two groups. But higher mean age was found by Koc et al. [21], Tarastchuk et al. [22]; which is probably due to increased life expectancy, geographical location and racial influences. The current study also showed a similar trend of sex ration like those of Koc et al. [21] and Tarastchuk et al. [22] with a male predominance in both groups.

In our study it was observed that the Group A had more patients with BMI ≥25 than Group B, which indicates that the risk of cardiovascular events rises with increasing body mass index (BMI). Tarastchuk et al. [22], Flegal et al. [23], Rosengren et al. [24], Willett et al. [25], Manson et al. [26], also reported the obesity as an independent risk factor for coronary artery disease (CAD) in both genders. Prospective epidemiological studies have revealed that central obesity is more relevant in CAD risk [27]. Gelber et al. [28] found WHtR had the strongest relation in association with incidence of CAD. In another study, Hong-Yan et al. [29] mentioned that waist to height ratio was the only anthrompometric index which had consistent association with cardiovascular risk factors. Schneider et al. [11] showed that the WHtR or WC may predict cardiovascular risk better than BMI or waist hip ratio (WHR), even though the differences are small. Ho et al. [17] have also shown that waist to height ratio (WHtR) is an effective abdominal obesity index in predicting the risk of diabetes and coronary heart diseases in the general population. All these results are similar to our observation of significantly high percentage of patients in Group A with higher BMI.

The findings of this current study indicate a week significant positive correlation with BMI but better significant positive correlation with Waist-to-height ratio in patients with significant Coronary artery disease. It is not surprizing that we have observed approximately 3 and 7 times more likelihood of developing significant coronary artery disease among patients with high BMI (≥25 kg/m^2^) and high WHtR (≥0.55). The relative risk of cardiovascular mortality at these levels of BMI has been reported to be 2 to 4 times higher in nearly all of the largest studies [30].

We had some limitations in this study. Firstly, the study was done in a single tertiary hospital and the sample size was relatively small. Secondly, the findings apply only to patients with Coronary artery disease and not necessarily to the general population and finally, the cross-sectional design of the study. A prospective cohort study can better compare the prognostic value of BMI and waist-to-height ratio in the natural care of coronary artery disease.

## Conclusion

This study was done to determine whether waist-to-height ratio better correlates with coronary artery disease (CAD) severity than body mass index (BMI). Coronary artery disease had significant positive correlation with both BMI and waist-to-height ratio, but more significant positive correlation was found between the severity of coronary artery disease and waist-to-height ratio. In addition, this study shows that waist-to-height ratio can be used as a risk factor of coronary artery disease.

### Ethics

The study protocol was approved by institutional ethical committee of Dhaka Medical College Hospital.

### Data sharing

There is no other unpublished data to share.

## Competing interest

The authors declare that they have no competing interests.

## Authors’ contribution

KMNS, AWC and HILRK were involved in planning, consultation and data collection for this study. ATM HH was involved in data analysis and writing the manuscript. The rest were involved in consultation and data collection. All the authors have read and approved the final version of the manuscript.
